# Choroidal and Macular Thickness in Eyes with Amblyopia

**DOI:** 10.14744/bej.2021.52714

**Published:** 2021-12-17

**Authors:** Rengin Aslihan Kurt, Sezin Akca Bayar, Zeynep Eylul Ercan, Eylem Yaman Pinarci, Mustafa Agah Tekindal, Sibel Oto

**Affiliations:** 1 Department of Ophthalmology. Baskent University Istanbul Health Application Research Center. Istanbul. Turkey; 2 Department of Ophthalmology. Baskent University Faculty of Medicine. Ankara. Turkey; 3 Department of Biostatistics. Baskent University Faculty of Medicine. Ankara. Turkey

**Keywords:** Amblyopia, choroidal thickness, enhanced depth imaging optical coherence tomography

## Abstract

**Objectives:**

This study was performed to assess and analyze the retinal and choroidal thickness in amblyopic eyes using spectral-domain optical coherence tomography and enhanced depth imaging optical coherence tomography.

**Methods:**

A total of 67 children with amblyopia and 31 age- and gender-matched healthy non-amblyopic control subjects were enrolled in the study. The 67 amblyopic children were divided into 3 groups: hypermetropic amblyopia (Group 1). microesotropia (Group 2). and myopic anisometropia (Group 3). All of the participants underwent a detailed ophthalmologic examination and orthoptic assessment. The subfoveal choroidal thickness was measured at 500-. 750-. 1000-. and 15000-micron intervals nasally and temporally. Central macular thickness was also measured in the subfoveal. parafoveal inferior. parafoveal superior. parafoveal nasal. and parafoveal temporal superior. inferior. nasal. and temporal quadrants. All of the parameters of the amblyopic eyes. fellow eyes. and control eyes were compared.

**Results:**

In all. 34 female and 33 male patients were studied. The mean age was 8.6±2.8 years (range: 5–12 years). Comparison of the choroidal thickness and macular thickness measurements between the amblyopic and non-amblyopic eye of the same patient within each group revealed no statistically significant differences. Comparison of the findings in the amblyopic eyes of Group 1 and Group 3 with the control group revealed that the choroidal thickness measurements were smaller in the amblyopic eyes in all quadrants. however. only the nasal quadrant measurements demonstrated a statistical significance. The subfoveal macular thickness value was also smaller in both groups when compared with the control eyes.

**Conclusion:**

Our results indicated that amblyopia. whether anisometropic or microtropic. did not seem to significantly affect choroidal thickness.

## Introduction

The most common cause of unilateral vision impairment in children and young adults is ambly-opia. with a prevalence of 0.72–3.29% (1). Amblyopia may be secondary to strabismus. aniso-metropia or deprivation (2). It is very well known that amblyopia is related to the changes in vis-ual cortex however changes in retina and choroid had been investigated by many different au-thors and varying results had been achieved (3). In the past decade. choroidal imaging has emerged with the advent of advanced imaging technologies. especially with the use of enhanced depth imaging optical coherence tomography (EDI-OCT) (4). EDI-OCT has enabled in vivo cross-sectional choroidal imaging and accurate quantitative analysis of choroidal thickness. Be-sides retina and choroid morphology in healthy children. the effect of amblyopia on retinal and choroidal morphology has been an area of interest (5-7).

## Methods

Patients aged between 5 and 12 years. who were amblyopic due to anisometropia or microtropia were included in the study group. The control group consisted of age and gender matched healthy non-amblyopes. This study was approved by Başkent University Institutional Review Board and Ethics Committee (KA13/58). The research adhered to the tenets of the Declaration of Hel-sinki. and a detailed written informed consent was obtained before each individual’s participa-tion in the study.

The participants underwent full ophthalmologic and orthoptic examination. Sixty-seven patients with amblyopia were analysed in three different subgroups. Group 1 (n=33. 49.2%) included hypermetropic ambliyopic children. Group 2 (n=23. 34.3%) included children with microeso-tropia and Group 3 (n=11. 16.4%) included myopic anisometropic children. Group 4 (n=31) consisted of the control group which had uncorrected visual acuity of 0.1 LogMAR (logarithm of minimum angle) in each eye and normal ocular findings. Anisometropia was defined as 1.5 D and more difference between amblyopic and non-amblyopic eyes and microtropia was ac-cepted as 8 PD and less squint. Patients with more than 10 PD manifest eso/exotropia or higher refractive error than 5 D were excluded.

All OCT scans were performed at the same time of the day. in the morning. to avoid diurnal fluctuations. Choroidal thickness measurements were performed by the same experienced tech-nician using a high speed and high-resolution Fourier domain-OCT device (λ=840 nm. 26.000 A-scans/s.. and 5 μm axial resolution). Optovue RTVue software version 3.5 (Optovue Inc.. Fremont. CA). The scan pattern was the retina cross line. consisting of 2 orthogon ally oriented 6-mm lines which contains 1024 A-scans. By automatically inverting the image. the chorioreti-nal interface became adjacent to the zero delay. The retina crossline scan has 32 frames aver-aged. 16/direction. without tracking (8).

**Figure 1.**
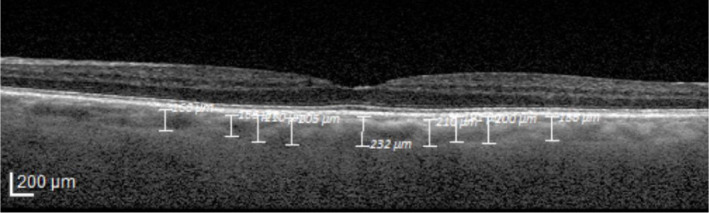
Choroidal thickness measurement in enhanced depth imaging optical coherence tomography.

Choroidal thickness was measured perpendicularly from the outer edge of the retinal pigment epithelium to the choroid-sclera boundary at the fovea and at 5 more points which are located at respectively. 500 μ nasal to the fovea. 1000 μ nasal to the fovea. 500 μ temporal to the fovea. 1000 μ temporal to the fovea and 1500 μ temporal to the fovea (Fig. 1). Choroidal thickness measurements were made by two masked physicians (SAB and EE). The average of the two measurements was taken; the differences between readings of the masked physicians were found to be within 10% of the mean.

### Statistical Analysis

The results of tests were expressed as the number of observations (n). mean±standard devia-tion. median and min-max values. The results of the homogenity (Levene’s Test) and normality tests (Shapiro Wilk) were used to decide which statistical methods to apply in the comparison of the study groups. Normally distributed and with homogeneous variances groups were com-pared two groups by Student’s t-test. Paired t-test and compared three or more groups by Anal-ysis of Variance. According to those tests results parametric test assumptions were not available for some variables. so the comparisons of two independent groups were performed by Mann-Whitney U test. comparisons of two dependent groups by Willcoxon test and three independent groups were performed by Kruskal Wallis test. Multiple comparison tests. the adjusted Bonfer-roni test was used. All statistical analyses were performed with the Statistical Package for the Social Sciences (SPSS) software (SPSS Ver. 17.0; SPSS Inc.. Chicago IL. USA). P<0.05 was considered statistically significant.

## Results

Of 67 patients 34 were female and 33 were male. The mean age was 8.6±2.8 (5–12) years. The mean best corrected visual acuity of amblyopic eyes was 0.23±0.09 (0.04–0.4) LogMAR in Group 1. 0.18±0.33 (0.09–1.3) LogMAR in Group 2. and 0.21±0.12 (0.1–0.8) LogMAR in Group 3. The mean best corrected visual acuity of non-amblyopic eyes was 0.02±0.04 (0–0.15) LogMAR. 0.02±0.06 (–0.07–0.09) LogMAR and 0.02±0.05 (0–0.09) LogMAR re-spectively. Spheric equivalent difference in between eyes was 1.75±1.81 (0.75–5.0) in Group 1. 0.99±0.87 (0–3.25) in Group 2 and 1.62±0.87 (1.50–3.62) in Group 3. Spheric equivalent difference in diopters was 1.75±1.81 (0.75–5.0) in Group 1. 0.99±0.87 (0–3.25) in Group 2 and 1.62 ± 0.87 (1.50–3.62) in Group 3 and 0.13±(0–0.75) in Group 4. Anisometropia in di-opters was 2.1±0.76 (1.50–4.50) in Group 1. 1.3±0.56 (0.50–3.25) in Group 2. 1.62±0.87 (1.50–3.62) in Group 3 and 0.23±(0–0.75) in Group 4 (Table 1).

**Table 1.** Demographic features of the study groups

**Groups**	**Hypermetropic Anisometropia Group 1**	**Micro-esotropica Group 2**	**Myopic Anisometropia Group 3**	**Control Group 4**
BCVA (Log-MAR) of the	0.23±0.09	0.18±0.33	0.21±0.12	0.0
amblyopic eye	(0.04–0.4)	(0.09–1.3)	(0.1–0.8)	
BCVA (Log-MAR) of the	0.02±0.04	0.02±0.06	0.02±0.05	0.0
non-amblyopic eye	(0–0.15)	(-0.07–0.09)	(0–0.09)	
Difference of SE values	1.75±1.81	0.99±0.87	1.62±0.87	0.13±
	(0.75–5.0)	(0–3.25)	(1.50–3.62)	(0–0.75)
Anisometropia (in Diopters)	2.1±0.76	1.3±0.56	1.62±0.87	0.23±
	(1.50–4.50)	(0.50–3.25)	(1.50–3.62)	(0–0.75)
Interocular line difference	3.8±	2.5±	2.4±	0
	(2–8)	(2–6)	(2–5)	

All values were shown as mean±standard deviation (range). BCVA: Best corrected visual acuity; SE: Spherical equivalent.

Choroidal thickness and macular thickness measurements were compared within each group between the ambliyopic and non-ambliyopic eye of the same patient. There were no statistically significant difference in any of the groups (Tables 2–4).

**Table 2.** Comparison of choroidal and macular thickness between amblyopic and non-amblyopic eyes in Group 1

**CT**	**Foveal**	**Nasal 500μ**	**Nasal 750μ**	**Nasal 1000μ**	**Nasal 1500μ**	**Temporal 500μ**	**Temporal 750μ**	**Temporal 1000μ**	**Temporal 1500μ**
GROUP-1	336.33	320.15	311.78	310.78	299.81	326.75	331.81	330.57	327.30
Amblyopic									
GROUP-1	350.75	331.03	325.75	321.03	305.69	331.72	336.30	334.96	334.12
Non-amblyopic									
p-value	0.376	0.492	0.364	0.506	0.690	0.787	0.774	0.785	0.654
**MT**	**Foveal**	**Parafoveal superior**	**Parafoveal inferior**	**Parafoveal nasal**	**Parafoveal temporal**	**Superior**	**Inferior**	**Nasal**	**Temporal**
GROUP-1	252.46	314.37	319.00	320.50	306.59	289.93	281.96	304.28	272.21
Amblyopic									
GROUP-1	251.21	312.87	313.62	317.84	304.50	286.46	276.12	298.53	266.50
Non-amblyopic									
p-value	0.713	0.584	0.068	0.104	0.068	0.383	0.155	0.153	0.234

Choroidal and macular thickness measurements did not show any statistically significant difference in between amblyopic and non-ambliyopic eyes in hypermetropic anisometropic group (p>0.05. for all). CT: Choroidal thickness; MT: Macular thickness.

**Table 3.** Comparison of choroidal and macular thickness between amblyopic and non-amblyopic eyes in Group 2

**CT**	**Foveal**	**Nasal 500μ**	**Nasal 750μ**	**Nasal 1000μ**	**Nasal 1500μ**	**Temporal 500μ**	**Temporal 750μ**	**Temporal 1000μ**	**Temporal 1500μ**
GROUP-2	380.00	352.13	353.21	348.91	342.04	364.34	358.95	357.56	353.08
Amblyopic									
GROUP-2	367.78	351.47	348.13	345.47	331.00	343.30	350.56	351.13	349.69
Non-amblyopic									
p-value	0.364	0.962	0.740	0.816	0.455	0.189	0.603	0.764	0.886
**MT**	**Foveal**	**Parafoveal superior**	**Parafoveal inferior**	**Parafoveal nasal**	**Parafoveal temporal**	**Superior**	**Inferior**	**Nasal**	**Temporal**
GROUP-2	249.00	316.13	311.66	317.45	303.68	284.59	273.45	286.54	269.09
Amblyopic									
GROUP-2	241.04	311.86	310.43	314.82	303.17	285.21	274.04	297.00	269.56
Non-amblyopic									
p-value	0.266	0.299	0.722	0.451	0.751	0.977	0.988	0.480	0.872

Choroidal and macular thickness measurements did not show any statistically significant difference in between amblyopic and non-ambliyopic eyes in microesotropic group (p>0.05. for all). CT: Choroidal thickness; MT: Macular thickness.

**Table 4.** Comparison of choroidal and macular thickness between amblyopic and non-amblyopic eyes in Group 3

CT	Foveal	Nasal 500μ	Nasal 750μ	Nasal 1000μ	Nasal 1500μ	Temporal 500μ	Temporal 750μ	Temporal 1000μ	Temporal 1500μ
GROUP-3	336.64	327.36	315.82	309.64	293.09	332.27	329.09	320.55	320.00
Amblyopic									
GROUP-3	333.91	325.91	314.45	303.18	291.45	323.36	322.55	326.00	309.09
Non-amblyopic									
p-value	0.917	0.948	0.934	0.737	0.93	0.675	0.78	0.767	0.517
**MT**	**Foveal**	**Parafoveal superior**	**Parafoveal inferior**	**Parafoveal nasal**	**Parafoveal temporal**	**Superior**	**Inferior**	**Nasal**	**Temporal**
GROUP-3	247.90	316.90	315.10	319.00	305.00	276.10	264.20	301.00	257.90
Amblyopic									
GROUP-3	243.90	320.20	310.50	316.60	290.60	279.90	266.30	296.90	262.40
Non-amblyopic									
p-value	0.276	0.613	0.22	0.347	0.204	0.229	0.376	0.23	0.507

Choroidal and macular thickness measurements did not show any statistically significant difference in between amblyopic and non-ambliyopic eyes in myopic anisometropic group (p>0.05. for all). CT: Choroidal thickness; MT: Macular thickness.

Choroidal thickness measurements of the amblyopic eyes in each group was compared to the control group separately. In group 1 and group 3 choroidal thickness measurements were thin-ner than the control group in all quadrants however only nasal quadrant measurements revealed statistical significance (p=0.045 for Group 1 and p=0.043 in Group 3). Choroidal thickness measurements of Group 2 did not show any statistical significant difference when compared to the control group (Table 5).

**Table 5.** Comparison of choroidal thickness in amblyopic eyes

**CT**	**Foveal**	**Nasal 500μ**	**Nasal 750μ**	**Nasal 1000μ**	**Nasal 1500μ**	**Temporal 500μ**	**Temporal 750μ**	**Temporal 1000μ**	**Temporal 1500μ**
GROUP-1									
Ambliyopic	336.33	320.15	311.78	310.78	299.81^*^	326.75	331.81	330.57	327.30
GROUP-2									
Ambliyopic	380.00	352.13	353.21	348.91	342.04	364.34	358.95	357.56	353.08
GROUP-3									
Ambliyopic	336.64	327.36	315.82	309.64	293.09^*^	332.27	329.09	320.55	320.00
CONTROL	377.45	348.54	339.75	335.74	324.88	350.22	355.48	351.69	347.33
p-value	>0.05	>0.05	>0.05	>0.05	^*^0.045. 0.043	>0.05	>0.05	>0.05	>0.05

^*^CC measurements in the amblyopic eye in hyperopic and myopic anisometropia were thinner in all quadrants than the control group. but only nasal 1500 μ was found to be statistically thin-ner than the control group (p=0.045. p=0.043); microtropic group measurements were not different with the control group (p>0.05) CT: Choroidal thickness.

Macular thickness measurements of the amblyopic eyes were compared to the control group. Macular subfoveal thickness was statistically significantly thinner in group 1. 2 and 3 in com-parison to the control group (p=0.00). Inferior quadrant central foveal thickness was found to be thinner in group 3 when compared to the control group (p=0.041) (Table 6).

**Table 6.** Comparison of macular thickness in amblyopic eyes

**MT**	**Foveal**	**Parafoveal superior**	**Parafoveal inferior**	**Parafoveal nasal**	**Parafoveal temporal**	**Superior**	**Inferior**	**Nasal**	**Temporal**
GROUP-1									
Amblyopic	252.46^*^	314.37	319.00	320.50	306.59	289.93	281.96	304.28	272.21
GROUP-2									
Amblyopic	249.00^*^	316.13	311.66	317.45	303.68	284.59	273.45	286.54	269.09
GROUP-3									
Amblyopic	247.90^*^	316.90	315.10	319.00	305.00	276.10	264.20^**^	301.00	257.90
CONTROL	308.08	314.48	315.54	318.20	307.67	284.24	274.35	298.54	267.79
p-value	^*^0.00	>0.05	>0.05	>0.05	>0.05	>0.05	^**^0.041	>0.05	>0.05

^*^Macular subfoveal thickness was found to be thinner in amblyopic eyes in all three groups than in the control group (p=0.00). ^**^Lower quadrant central foveal thickness was found to be thinner in amblyopic eyes in myopic anisometropia (p=0.041). MT: Macular thickness..

## Discussion

In this study our aim is to compare the choroidal and macular thickness measurements in am-blyopic eyes with the fellow eye and the healthy control group. Many different studies by dif-ferent authors have been published on this topic until today and as a result there are controver-sies in the literature regarding the choroidal thickness and macular thickness changes in ambly-opic eyes.

Very first studies that analysed macular thickness in amblyopia are from early 2000 s and many different results have been achieved (9). In a meta-analyses including 28 studies with 408 pa-tients. many controversial results have been found however the authors concluded that amblyo-pia is associated with foveal and macular thickness increase (10). Similar to the results of Li’s me-ta-analyses from 2015. more recent studies of Rajawi et al. (11). Kasem et al. (12). Kuhli-Hattenbach et al. (13) revealed increased central macular thickness in amblyopic eyes. Taskiran Comez et al. (14) and three more studies did not show any statistically significant difference in amblyopic and fel-low eyes (15-17). In our study. no statistically significant difference in macular thickness was found between the amblyopic and fellow eyes which is compatible with the recent studies. We found that macular subfoveal thickness was statistically significantly thinner in all groups when compared to the control group. This finding was thought to be due to the morphological effect of amblyopia.

Xu et al. (18) found that there were no differences in the thickness of the foveal and the retinal nerve fiber layers between eyes with esotropic amblyopic and the normal fellow eyes. Pang et al. (19) analyzed 31 children with unilateral high myopia. Their results revealed that amblyopic children with unilateral high myopia have a thicker fovea and thinner inner and outer macula in the am-blyopic eye. However. these findings were thought to be due to the morphological effect of amblyopia.

It is well known that choroid has a role in emmetropization however imaging of the choroid and accurate measurements could only be possible within the last decade with the progress in poste-rior segment imaging. Since resolution of the spectral domain OCT below the level of retinal pigment epithelium is very low. EDI-OCT offers the required technology for choroidal imaging.

Vincent et al. (20) analysed the eyes of 22 non-amblyopic myopic anisometropes and found that subfoveal choroid was thinner in the amblyopic eyes and this finding was correlated with the axial length. Araki et al. (21) examined 31 patients with hyperopic anisometropic amblyopia. 15 patients with strabismic amblyopia without anisometropia and 24 age-matched controls. Their results showed that the anisometropic amblyopia group had thicker choroids than that of fellow eyes and control group. It is hypothesised that the difference in choroidal thickness in myop-ic amblyopes and hyperopic amblyopes may be due to the axial length alterations. Despite these controversial results a meta-analysis of 11 clinical trials with 449 patients showed that the cho-roid was found to be thicker in the amblyopic eyes than that in the fellow and control eyes (22). Similarly in a recent study of Zha et al. (23) with 31 children. choroid was found significantly thick-er in subfoveal area. 1 mm nasal and 1 mm superior to the fovea in amblyopic eyes than control eyes. In the study of Hansen et al. including 20 children with amblyopia. subfoveal choroidal thicknesses was found to be higher in amblyopic eyes when compared to fellow eyes and healthy controls (15). In a study of Niyaz et al. (24). 90 patients with different categories of amblyopia such as anisometropic. strabismic and mixed were analysed and increased choroidal thickness was found in anisometropic group compared to fellow and control eyes.

Karaca et al. (25) showed that macular choroidal thickness was thicker than healthy controls both in amblyopic and non-amblyopic eyes. They hypothesized that this finding can indicate bilateral emmetropization delay implying that amblyopia influences both eyes’ visual feedback. Since the subfoveal choroidal thickness was found to be higher in amblyopic eyes and fellow eyes in a study of Xu et al. (26). their results suggested thicker choroid might be a used as a diagnostic pa-rameter for amblyopia.

However in our study there was no statistically significant difference in choroidal thickness measurements between the ambliyopic and non-ambliyopic eyes. In our study choroidal thick-ness measurements of the amblyopic eyes in each group was compared to the healthy control group separately as well. In hyperopic anisometropic group and myopic anisometropic group choroidal thickness measurements were thinner than the control group in all quadrants however only nasal quadrant measurements revealed statistical significance.

Nishi et al. (27) analysed the choroidal structure in amblyopic children by using binarization of opti-cal coherence tomography images. The total choroidal area and luminal/stromal ratio in the am-blyopic eyes was significantly larger than that of the fellow eyes. They speculated that the lumen/stroma ratio increase may be due to the immature development of these eyes. Similarly Baek et al. (28) analysed choroidal vascularity in eyes of 32 hyperopic amblyopic children and 38 healthy controls. They observed a positive correlation between choroidal thickness and cho-roidal vascularity in healthy eyes. However in amblyopic eyes a negative correlation was ob-served which was thought to show decreased blood supply of the choroid and outer retina in amblyopic eyes. While several factors may affect subfoveal choroidal thickness. choroidal vascularity index (CVI) remained unaltered. implying that CVI is a more reliable indicator of choroidal disorders (29).

In our study comparison of choroidal thickness in the amblyopic and non-ambliyopic fellow eyes did not show any statistical significance. This finding suggested that amblyopic eyes have a metabolic activity which is as high as the fellow eye of the same patient. Mohan et al. (30) studied retinal vascular oxygen saturation in amblyopic eyes and compared them to unaffected fellow eyes and healthy control group. Controversial to our results. they found that amblyopic eyes showed higher mean oxygen saturations than the fellow eyes which was thought to be neuronal activity alterations. While interpreting the results of all these various studies. it is important to remember that subfoveal choroidal thickness is influenced by different factors like age. ethnici-ty. intraocular pressure and axial length (31).

Choroidal thickness may change with treatment and it had been shown by many different authors. Hashimoto et al. (32) showed that both choroidal blood flow increased and choroidal thickness decreased in the amblyopic eyes with treatment in two anisohypermetropic amblyopiacases. In a series of Nishi et al. (33) with 24 anisohypermetropic amblyopic children showed optical correction resulted in choroidal thinning in the amblyopic eyes and fellow eyes. Araki et al. (34) investigated the effect of amblyopia treatment on macular choroidal thickness in 13 patients. Controversial to Nishi and Hashimoto. their results revealed that despite the increase in visual acuity. treatment did not cause any decrease in the choroidal thickening.

In conclusion although our study has a limitation of relatively small sample size. we believe that our results have scientific value. Prospective studies with larger patient numbers can enlighten the choroidal and macular structural changes in amblyopia.

### Disclosures

**Ethics Committee Approval:** This study was approved by Başkent University Institutional Review Board and Ethics Committee (KA13/58).

**Peer-review:** Externally peer-reviewed.

**Conflict of Interest:** None declared.

**Authorship Contributions:** Involved in design and conduct of the study (SAB, SO); preparation and review of the study (RAK, SAB); data collection (ZEE, EYP); and statistical analysis (MAT).
